# TLR3 and TLR9 Agonists Improve Postexposure Vaccination Efficacy of Live Smallpox Vaccines

**DOI:** 10.1371/journal.pone.0110545

**Published:** 2014-10-28

**Authors:** Tomer Israely, Sharon Melamed, Hagit Achdout, Noam Erez, Boaz Politi, Trevor Waner, Shlomo Lustig, Nir Paran

**Affiliations:** Department of Infectious diseases, Israel Institute for Biological Research, Ness-Ziona, Israel; Uniformed Services University, United States of America

## Abstract

Eradication of smallpox and discontinuation of the vaccination campaign resulted in an increase in the percentage of unvaccinated individuals, highlighting the need for postexposure efficient countermeasures in case of accidental or deliberate viral release. Intranasal infection of mice with ectromelia virus (ECTV), a model for human smallpox, is curable by vaccination with a high vaccine dose given up to 3 days postexposure. To further extend this protective window and to reduce morbidity, mice were vaccinated postexposure with Vaccinia-Lister, the conventional smallpox vaccine or Modified Vaccinia Ankara, a highly attenuated vaccine in conjunction with TLR3 or TLR9 agonists. We show that co-administration of the TLR3 agonist poly(I:C) even 5 days postexposure conferred protection, avoiding the need to increase the vaccination dose. Efficacious treatments prevented death, ameliorated disease symptoms, reduced viral load and maintained tissue integrity of target organs. Protection was associated with significant elevation of serum IFNα and anti-vaccinia IgM antibodies, modulation of IFNγ response, and balanced activation of NK and T cells. TLR9 agonists (CpG ODNs) were less protective than the TLR3 agonist poly(I:C). We show that activation of type 1 IFN by poly(I:C) and protection is achievable even without co-vaccination, requiring sufficient amount of the viral antigens of the infective agent or the vaccine. This study demonstrated the therapeutic potential of postexposure immune modulation by TLR activation, allowing to alleviate the disease symptoms and to further extend the protective window of postexposure vaccination.

## Introduction

Variola virus (VARV), the causative agent of smallpox claimed the life of hundreds of millions throughout history prior to the World Health Organization's (WHO) declaration, about three decades ago, that smallpox has been eradicated. This was achieved by a world-wide vaccination campaign utilizing vaccine strains of vaccinia virus (VACV) and other closely related family members [1]. Disease eradication allowed for the gradual discontinuation of the vaccination campaign, resulting in an increase in the percentage of unimmunized individuals worldwide. The growing concern that smallpox might emerge as a consequence of accidental or intentional release of VARV, emphasizes the need for postexposure (p.e.) countermeasures that will be effective, and can be easily and rapidly applied for mass vaccination [2].

A relatively long incubation period of about 7–14 days in human smallpox opens a window of several days for optional p.e. intervention [3]. Anecdotal reports of protection by p.e. vaccination indicate that there is a potential benefit for this treatment [4]. Using ectromelia virus (ECTV), the causative agent of mousepox, we along with others, demonstrated the similarity of various aspects of the disease between mousepox and human smallpox substantiating the relevance of this animal model to simulate various aspects of the human disease and measures of protection [5]–[9]. In this animal model, p.e. treatments were examined using different vaccine strains [8], antiviral drugs [7], [10], [11] and antibodies [12]. In agreement with the historical human studies, vaccination of mice with VACV conferred solid p.e. protection if given up to 3 days p.e. at a high vaccination dose [8]. Comparable results were obtained with MVA, a highly attenuated vaccine. Both confer p.e. protection yet vaccination is the only method with approved efficacy in eradication of smallpox while ensuring both short and long term immunity.

Protection by p.e. active vaccination requires the induction of rapid and potent, yet durable immune response. Despite the fairly long incubation period, evasion of host immunity by these virulent viruses and the rate of developing immunity (innate and adaptive) hampers the efficacy of p.e. vaccination and restrict the protection window to the first 3–4 days p.e. We have previously shown, that at least in the mouse-ECTV animal model, efficacy of p.e. protection by active vaccination is strongly affected by the vaccination dose (1×10^8^ pfu of either VACV-Lister or MVA are preferable) [8]. Whether this is also relevant in humans has not been determined, yet, the logistical consequences of increasing the vaccination dose (for VACV) calls for an alternative mechanism to rapidly induce potent immunity. One possible way to increase the potency of the immune response is by co-administration of adjuvants with the vaccine. Preexposure co-administration of adjuvants and vaccines is widely used as an approved protocol to enhance the immune response to non-replicative antigens: either purified proteins, DNA, or whole inactivated viruses or bacteria [13].

The TLRs family, also known as pattern recognition receptors (PRRs), includes in humans 10 members (TLR1-10) of transmembrane receptors that recognize general conserved patterns molecules (e.g. foreign DNA, RNA, liposacharides and lipoproteins) [14], [15]. Signal transduction following receptor activation is mediated by adaptor molecules such as MyD88, TRIF, IRF3, IRF7 and IKKs that trigger an intracellular cascade leading to activation of Type I IFN response and/or secretion of inflammatory cytokines and enhancement of the innate immunity confronting the invasive pathogen [16]. These receptors and their adaptors play an essential role in the activation of innate and subsequently adaptive immunity. The specificity of TLR 3, 7, 8, and 9 to nucleic acids (DNA and RNA), and their endosomal localization allows for detection of invading pathogens and to the development of immunity to viral infections [17]. TLR9 detects unmethylated CpG motifs present at high frequency in microbial rather than mammalian DNA [18], [19] and is expressed mainly by plasmacytoid DCs (pDCs), B cells, monocytes and mature macrophages, while TLR3 (expressed by DCs, B cells, epithelial and endothelial cells) is triggered by double-stranded RNA (dsRNA), produced during replication of mainly RNA viruses [20]–[22]. The immunostimulatory effect of the TLR3 and TLR9 can be mimicked by synthetic analogs of dsRNA (i.e. poly(I:C)) and oligonucleotides (i.e. ODNs containing CpG motifs), respectively. Three major classes of CpG ODNs have been described [16], [23] of which we examined in this study an “A” type ODNs which trigger pDCs to produce IFNs and in so doing activates a T and NK cell response and a “B” type ODN, known as a strong modulator of B cell activation.

The role of TLRs in detection of bacterial and viral components and induction of immunity set the basis for the development and use of TLR agonists as molecular adjuvants to improve the immune response of vaccines [23]. Examples include the evaluation of a TLR3 ligand in influenza [24]; and human papillomavirus vaccines (HPV, [25]), a TLR5 ligand in influenza vaccine [26], [27], a TLR9 ligand in malaria vaccine [28], [29] and a TLR4 ligand in Hepatitis B virus vaccine [30]. Some of these TLR ligands are in stages of preclinical and clinical trials while the AS04 (MPLA, TLR4 agonist formulated with alum) is approved for clinical use, as an adjuvant for HPV vaccine in Europe [31].

The genome of poxviruses is a large dsDNA (approximately 180–200 Kbps) encoding for about 200 ORFs. Pre-treatment with a protein-based smallpox vaccine formulated with CpG-ODNs and alum [32] or QS-21 [33] adjuvants, improved immunogenicity and protection conferred by the recombinant non-adjuvanted proteins. GM-CSF or *E. coli* heat-labile enterotoxin (LT) increased protective antibody responses of a 4pox gene-based vaccine in mice [34]. Interestingly, also intranasal vaccination with inactivated vaccinia virus formulated with poly(I:C), conferred higher antibody titer than the non-adjuvanted vaccine, suggesting that sensing foreign nucleic acids induces anti-viral immune responses, regardless of the exact composition of their genetic material [35].

Increased morbidity in poxvirus infected TLR9 deficient mice highlighted the importance of TLR9 in protection against poxvirus infections and development of poxvirus specific immunity [36]. Additionally, as part of their immune evasion strategy, poxviruses encode several virulence proteins (N1, A52, A46) known to inhibit intracellular signaling by a range of TLRs [37]. The important role of TLRs in acquirement of rapid immune response highlights the potential benefit of p.e. TLR activation.

Regardless of the specificity of the TLR ligand, so far, these ligands were formulated with proteins or inactivated viruses and used as adjuvants in vaccines given in a pre-exposure scenario. In the present study we evaluated the contribution of co-administered TLR-agonists to the protective immune response elicited by live vaccines (VACV-Lister or MVA) in a p.e. scenario. We further show that a TLR3 agonist conferred protection even without the vaccines but this required sufficient amount of viral antigens (of the infective virus). Such treatment will enable coverage of both short and long term immunity. To the best of our knowledge this is the first report on a work examining the use or applicability of adjuvants co-administered with live vaccines in a p.e. situation.

## Materials and Methods

### Cells and viruses

ECTV strain Moscow (ATCC VR-1374), VACV-Lister (Elstree; provided by the Israeli Ministry of Health) and MVA clonal isolate F6 at the 584^th^ CEF passage were propagated and tittered as described previously [8]. ECTV expressing firefly luciferase (ECTV-Luc) [38] was kindly provided by Prof. Luis Sigal, Research Institute of Fox Chase Cancer Center, Philadelphia, USA. Briefly, ECTV Moscow and ECTV-Luc were propagated in HeLa cells (ATCC-CCL-2) and titrated on BS-C-1 cells (ATCC-CCL-26). VACV-Lister was propagated on the chorioalantoic membranes of embrionated eggs and titrated on Vero cells (ATCC-CCL-81). MVA was propagated on secondary chicken embryo fibroblasts and titrated on BHK-21 cells (ATCC-CCL-10).

### Animal challenge experiments

General procedures for animal care and housing were followed in compliance with the regulations for animal experiments at the Israel Institute for Biological Research (IIBR). All experiment protocols (M-45-2011, M-22-2012, M-52-2012 and M-05-2013) were approved by the IACUC (Institutional Animal and Care Committee) of IIBR before commencement of the studies. All efforts were made to minimize animal suffering. The end-points were weight loss (25% of the initial weight in the infected untreated groups and 40% in the treated groups) and/or inability to respond to the righting reflex [39]. Animals that reached these predetermined end-points were humanely sacrificed by cervical dislocation. Mice were weighed every 1–3 days during the first two weeks and then every 2–5 days until the end of the experiment (day 25–30). Whenever mice reached a weight loss of more than 20% a daily based individual weigh was performed. All challenges and treatments were performed once on anesthetized mice (Ketamine 75 mg/kg, Xylazine 7.5 mg/kg in PBS). Non-infected untreated and infected untreated groups served in all experiments as controls. Placebo treatments were performed with PBS. The number of mice per group for each experiment is given in the tables or in the corresponding figure legends. Mice were housed on a 12 h light-dark cycle, with the dark cycle occurring from 7∶00 P.M. to 7∶00 A.M with *ad libitum* food and water supply. Mice were housed in a specific pathogen-free environment in individually ventilated cages (IVC). Prior to each experiment mice were randomly divided to experimental groups of 3–6 animals to achieve significant statistical data.

Female BALB/c mice (6–8 weeks old, 15–18 grams) were purchased from Charles River Laboratories, Margate, UK. Female C57BL/6j mice (6–8 weeks old, 15–18 grams) were purchased from Jackson Laboratory (JAX Mice, MA). Mice were acclimatized under supervision for a week prior to the experiment. For intranasal (i.n.) challenge, mice were anesthetized and ECTV (20 µl) was administered by instillation to one of the nostrils.

In the BALB/c experiments the challenge dose ranged from 4 to 20 ECTV LD_50_ (1 pfu = 1 LD_50_; total of 4 experiments) and in the C57BL/6j mice the challenge dose ranged from 2 to 3 ECTV LD_50_ (250 pfu = 1 LD_50_; total of 2 experiments). ECTV-Luc was used for bioluminescence study in BALB/c mice. Mice were infected with 2 i.n. LD_50_ (7 pfu = 1 i.n. LD_50_), left untreated (n = 5) or treated with poly(I:C) on day 3 p.e. (n = 3) and scanned on days 7, 8, 14 and 16 p.e.

Poly(I:C) (High molecular weight, InvivoGen, CA) and CpG oligonucleotides ODN 1826 and ODN 1585 (B and A type, respectively; InvivoGen, CA) were prepared according to the manufactures guidelines, aliquoted and froze (−20°C) for further use.

Vaccinations with VACV-Lister (i.d. tail scarification, 1×10^6^ pfu in 10 µl) or MVA (i.m., 1×10^8^ pfu in 50 µl) were carried out as described previously [7]. Poly(I:C) was administered s.c. at the base of the tail (100 µg in 100 µl). CPG-ODN 1826 and CPG-ODN 1585 were administered s.c. at the site of the i.d. tail scarification (50 µg in 10 µl). These TLR agonists were administered either alone or concomitantly with the vaccines.

In all cases, treatments were given to anesthetized mice once as a single treatment.

### Serum preparation and determination of viral load in mouse organs

Viral loads of ECTV in spleen, liver and lung were determined in mice 8 days p.e. as previously described [7]. Mice were bled from the tail vein prior to organ removal and the sera was separated by separation tubes (BD Microtainer, UK) following centrifugation (10,600 g) and kept in −20°C.

### Bioluminescence imaging

Live imaging was performed with an IVIS Lumina II system (Caliper LifeSciences, MA). D-Luciferin substrate (Caliper LifeSciences, MA) was injected intraperitoneally (i.p.) (150 µg/g body weight) 7 min prior to imaging. Mice were imaged under anesthesia with Ketamine 75 mg/kg, Xylazine 7.5 mg/kg in PBS. Mice were imaged on days 7, 8, 14 and 16 p.e. Images were collected for 1 or 40 s with binning factor of 4. Same region of interest (ROI) was used in all examined mice for calculation of signal intensity (from the chest through the hind limbs). Light emission was measured in photons/s/cm2/sr (photon flux). Acquisition and analysis were performed with Living Image Software, Version 4.2 (Calliper LifeSciences, Hopkinton, MA).

### Quantification of IFN-γ and IFN-α

Serum concentration of IFN-γ was measured 8 days p.e. using Quantikine mouse IFN-γ Immunoassay kit according to the manufacturer's instructions (R&D Systems, MN) and as previously described [7]. IFN-α concentrations in the serum (1 day after treatment or at the corresponding days p.e. in the infected untreated group) were measured using VeriKine mouse IFN-α ELISA kit according to the manufacturer's instructions (PBL InterferonSource, NJ).

### Determination of IgM antibody titer

Vaccinia specific IgM antibody titers were determined in mice from day 8 p.e. sera by ELISA. 96-well microtiter plates were coated over-night with VACV WR (1×10^7^ pfu/ml, 100 µl/well, 4°C). Virus was inactivated using 3.7% paraformaldehyde for 10 min at room temperature (50 µl/well for a final concentration of 2.4%) and followed by 3 washes in wash buffer (0.05% Tween 20 in PBS). Samples in a final dilution of 1∶200 in TSTA (50 mM Tris pH 7.6, 142 mM NaCl, 0.05% Azid, 0.05% Tween 20 and 2% BSA) were added to the plate for 1 h in 37°C following blocking with TSTA for 1 h at 37°C. Alkaline phosphatase conjugated goat anti-mouse IgM diluted 1∶1000 in TSTA (Jackson ImmunoResearch Laboratories, Inc. PA) was added after 3 washes and incubated for 1 hour at 37°C. *P-*nitrophenyl phosphate substrate (Sigma, MO) was added after 3 washes and optical density was measured (Spectramax 190 microplate reader, Molecular Devices, Sunnyvale, CA, O.D. of 405 nm) after 60 min incubation at room temperature. IgM values were determined by subtraction twice the value of buffer control well from the examined sample.

### Surface and intracellular cytokine staining

Mouse splenocytes were stained for extracellular markers followed by intracellular staining (ICS) using the Cytofix/Cytoperm containing monensin kit (BD Biosciences, NC) according to manufacturer instructions. The antibodies that were used were anti-CD3ε (clone 145-2C11), anti-CD8α (clone 53-6.7), anti-CD4 (clone RM4-5), anti DX5 (clone DX5) and anti IFNγ (clone XMG 1.2, all from e-Bioscience). Acquisition was performed with FACSCalibur flow cytometer (BD Biosciences, San Jose, CA) and analyzed with FlowJo 7.6 software (Tristar, CA). The total NK, CD3^+^CD4^+^ and CD3^+^CD8^+^ cell numbers in the spleen were determined by multiplying the percentage of each cell type by the total number of cells isolated from the organ.

### Histology and immunohistochemistry

Tissue samples (lung, spleen and liver) were fixed in 4% paraformaldehyde in PBS for 7–14 days, transfer to 70% ethanol, embedded in paraffin, serially sectioned (5-µm thickness) and stained with hematoxylin and eosin (H&E). For ECTV detection, sections following antigen retrieval (10 mM citrate buffer, 0.05% Tween-20, pH 6.0 for 20 min in 95–100°C), were treated according to the procedure recommended by the manufacturer using our in-house rabbit anti-ECTV antibody diluted 1∶250 in PBS +1% BSA (EnVision+ System-HRP (DAB) for use with rabbit primary antibodies, DAKO, Carpinteria, CA). CD45 staining was performed using biotinylated rat anti mouse CD-45 antibody (R&D systems, MN) following antigen retrieval. Cell and Tissue Staining Kit for rat, HRP-DAB system was used for signal enhancement (R&D systems, MN). Counter stain with hematoxylin was performed in all the slides.

### Data analysis

Kaplan-Meier survival plots were compared by the Cox-Mantel test. Morbidity analysis was performed as means of the area under the curve (AUC) in percent of individual weight at baseline as previously described [40]. Briefly, the AUC was weighted with the length of observation period starting the day of challenge (day 0) until the end of the experiment (day 25–30) or the day the animal died. The differences between groups were analyzed using student *t*-test. Mann-Whitney analysis was performed to compare viral load data between groups. The lethal dose of 50% for BALB/c and C57BL/6j mice was calculated according to Reed et. al. [41]. Reported *p* values <0.05 were considered significantly different. Throughout the manuscript average data is presented as mean value (±standard error of the mean (SEM)). For the IFNγ – viral load correlation linear regression was used while for the IFNγ – IgM correlation a nonlinear fit was drawn from the exponential growth curve. Statistics were performed using GraphPad Prism 5 for Windows (version 5.00, GrapPad Software Inc., San Diego, CA).

## Results

### Extending the therapeutic window of postexposure vaccination by co-administered TLR3 and TLR9 agonists

**Figure 1:**
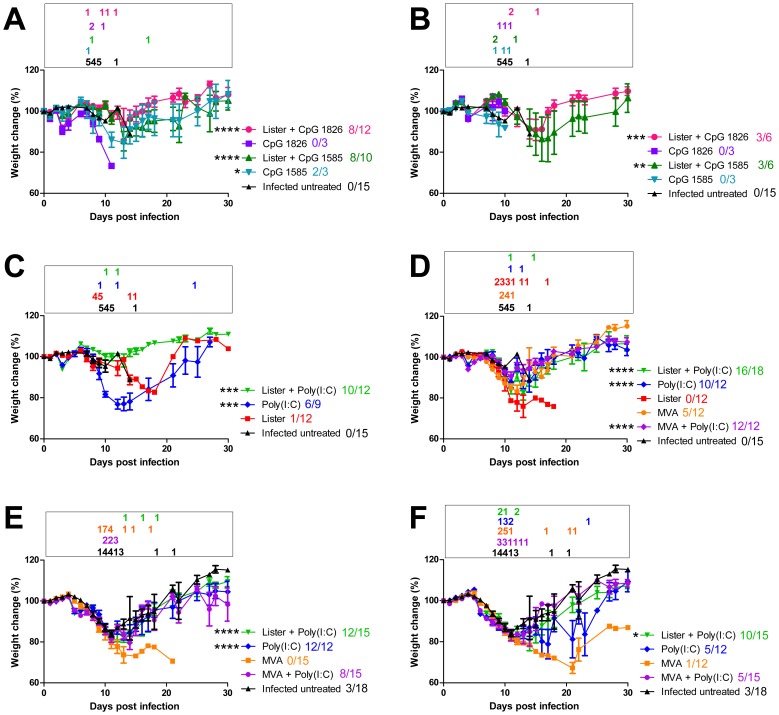
Morbidity based on weight change following post exposure (p.e.) treatments of BALB/c mice. Mice were infected with 4–20 i.n. ECTV LD_50_. (A, B) CPGs treatments with or without VACV-Lister on day 2 (A) and 3 (B) p.e. (C) Poly(I:C) treatments with or without VACV-Lister or VACV-Lister alone on day 2 p.e. (D) Poly(I:C) treatments with or without VACV-Lister or MVA and only vaccines treatments on day 3 p.e. (E, F) Poly(I:C) treatments with or without VACV-Lister or MVA and only MVA on day 4 (E) and 5 (F) p.e. Asterisk denote for significant difference in the area-under-the curve of weight changes along the entire experiment of the treated groups vs. the infected untreated group (* P<0.05, ** P<0.01, *** P<0.001, **** P<0.0001, *t*-test). Data collection for each treatment (weight change (mean, SE)) is indicated. Morbidity of Infected untreated mice from corresponding relevant experiments is shown. The number of mice succumbed to the infection in each time point is outlined color coded in a box above each graph. Survivals out of the total mice in each group are designated color coded next to the legend.

In order to evaluate the ability of TLR9 and TLR3 agonists to extend the therapeutic potential of postexposure vaccination we used an established mouse model of ECTV infection [7], [10], [11]. BALB/c mice infected with a lethal dose of ECTV exhibited signs of illness (weight loss (Figure 1 A), furred hair) starting day 7–8 p.e. and succumbed to infection within mean time to death (MTTD) of 11.3±3.4 days. Placebo treated mice (PBS) succumbed to infection similarly to the infected untreated group (MTTD of 10.7±1.2 and 11.2±2.7 for day 2 and 3 p.e. respectively). Under these conditions, vaccination with VACV-Lister did not protect the mice (Table 1, 8% on day 2 and 0% on day 3 p.e., Figure 1 C, D). Co-administration of the TLR9 agonists, CpG 1826 (B type) or CpG 1585 (A type) to the vaccine on days 2 or 3 p.e. conferred significant protection (Table 1, Figure 1 A, B). CpG 1585 administration without a vaccine conferred partial protection while CpG 1826 administration was not protective (Table 1, Figure 1 A, B).

**Table 1:** Survival Table – BALB/c mice.

Survival following treatment on:	Day 2 p.e.	Day 3 p.e.	Day 4 p.e.	Day 5 p.e.
Treatment				
Lister	8% _(1/12)_	0% _(0/12)_	N.D.	N.D.
Lister+CpG 1826	67% _(8/12)_ ****	50% _(3/6)_ **	N.D.	N.D.
Lister+CpG 1585	80% _(8/10)_ ****	50% _(3/6)_ *	N.D.	N.D.
CpG 1826	0% _(0/3)_	0% _(0/3)_	N.D.	N.D.
CpG 1585	67% _(2/3)_ *	0% _(0/3)_	N.D.	N.D.
Lister+poly(I:C)	83% _(10/12)_ ****	89% _(16/18)_ ****	80% _(12/15)_ ****	67% _(10/15)_ **
Poly(I:C)	67% _(6/9)_ *	83% _(10/12)_ ****	100% _(12/12)_ ****	42% _(5/12)_
MVA	N.D.	45% _(5/12)_	0% _(0/15)_	8% _(1/12)_
MVA+poly(I:C)	N.D.	100% _(12/12)_ ****	53% _(8/15)_ *	33% _(5/15)_

Infected untreated BALB/c mice i.n. infected with ECTV (survived/total (n)): 3/39 (8%) which are the sum of Exp.1 4LD_50_ (0/6); Exp.2 15LD_50_ (0/15); Exp.3 5LD_50_ (2/6); Exp.4 20LD_50_ (1/12).

*P<0.05, **P<0.01, ***P<0.001, ****P<0.0001 Log-rank (Mantel-Cox test) vs. infected untreated (of the relevant infected untreated group in the same experiments).

N.D. – not determined; p.e. – postexposure.

Next, we evaluated the protective effect of the TLR3 (RNA) agonist, poly(I:C). Co-administration of poly(I:C) with the vaccine prevented death in 83%, 89%, 80% or 67% of the mice treated on days 2, 3, 4 or 5, respectively (P<0.0001 for days 2, 3, 4 and P<0.01 for day 5 vs. infected untreated mice, Table 1). Interestingly, administration of poly(I:C) without the vaccine also prevented death (67%, 83% or 100% of the mice on days 2, 3 or 4 respectively (P<0.05 on day 2 and P<0.0001 on days 3 and 4 compared to infected untreated mice)), yet, statistically significant protection on day 5 was achieved only when poly(I:C) was combined with the vaccine. Similarly to the TLR9 ligands, mortality rates reflected the extent of morbidity. Poly(I:C) treatment with VACV-Lister, given on day 2, 3, 4 or 5, delayed and significantly reduced the extent of the weight loss (P<0.0001 for days 2, 3, 4 and P<0.05 for day 5, Figure 1 C–F)).

Unlike VACV-Lister, MVA does not replicate in mammalian hosts which on the one hand makes MVA a safe vaccine but on the other hand necessitates a 100 fold higher vaccine dose. To evaluate the efficacy of co-administration of poly(I:C) and MVA, BALB/c mice were p.e. vaccinated with MVA (1×10^8^ pfu i.m.) with or without poly(I:C) (s.c.). MVA vaccination 3 days p.e. protected 45% of the mice while vaccination on days 4 or 5 no longer conferred protection (Table 1, 0% on day 4 and 8% on day 5, Figure 1 D–F). Co-administration of poly(I:C) and MVA improved protection to 100%, 53% and 33% on days 3, 4 and 5, respectively (P<0.0001 for day 3, P<0.05 for day 4 and P = 0.3 for day 5 p.e. compared to the infected untreated). Protective co-administration of MVA and poly(I:C) alleviated the morbidity compared to unprotected mice (P<0.0001, P = 0.08 and P = 0.4 for days 3, 4 and 5 respectively; Figure 1D–F ).Co-administration of poly(I:C) and MVA had no advantage over poly(I:C) (Table 1, Figure 1).

Similar experiments were performed with C57BL/6j mice that differ from BALB/c in the sensitivity to ECTV infection (i.n. LD_50_ in BALB/c is 1 pfu while in C57BL/6j is 250 pfu). Mice were challenged with 2–3 i.n. LD_50_ of ECTV (MTTD of infected untreated: 11.2±1.9 days) and treated on days 0, 1 or 2 p.e. While VACV-Lister did not protect neither on day 0 nor day 1, the combination of the poly(I:C) and VACV-Lister conferred significant protection (55% and 45% survival on days 0 and 1, P<0.0001 in both cases compared to the infected untreated mice, Table 2). Co-administration of poly(I:C) and MVA, one or two days p.e., also improved the protection compared to that achieved by MVA alone (day 1 from 72% to 100%; day 2 from 18% to 55%). Protective co-administration of poly(I:C) and either VACV-Lister or MVA did not prevent the morbidity deterioration during the first 10 days p.e. but prevented further deterioration and death (Figure S1, P<0.01 for VACV-Lister+poly(I:C) and P<0.001 for MVA+poly(I:C) based on AUC analysis compared to infected untreated or only vaccinated). Administration of only poly(I:C) on day 1 p.e. protected 43% of the mice (P<0.0001 compared to infected untreated mice).

**Table 2:** Survival Table – C57BL/6j mice.

Survival following treatment on:	Day 0 p.e.	Day 1 p.e.	Day 2 p.e
Treatment			
Lister	0% _(0/11)_	0% _(0/9)_	N.D.
Lister+poly(I:C)	55% _(6/11)_***	45% _(5/11)_***	N.D.
MVA	N.D.	72% _(8/11)_***	18% _(2/11)_ *
MVA+poly(I:C)	N.D.	100% _(11/11)_***	55% _(6/11)_***
Poly(I:C)	22% _(2/9)_**	43% _(6/14)_***	27% _(3/11)_**

Infected untreated C57BL/6j mice i.n. infected with ECTV (survived/total (n)): Exp.1 2LD_50_ (0/3); Exp.2 3LD_50_ (0/8).

*P<0.01, **P<0.001, ***P<0.0001 Log-rank (Mantel-Cox test) vs. infected untreated (0/11).

N.D. – not determined; p.e. – postexposure.

### Correlation of effective treatments with reduced viral load in peripheral organs

**Figure 2:**
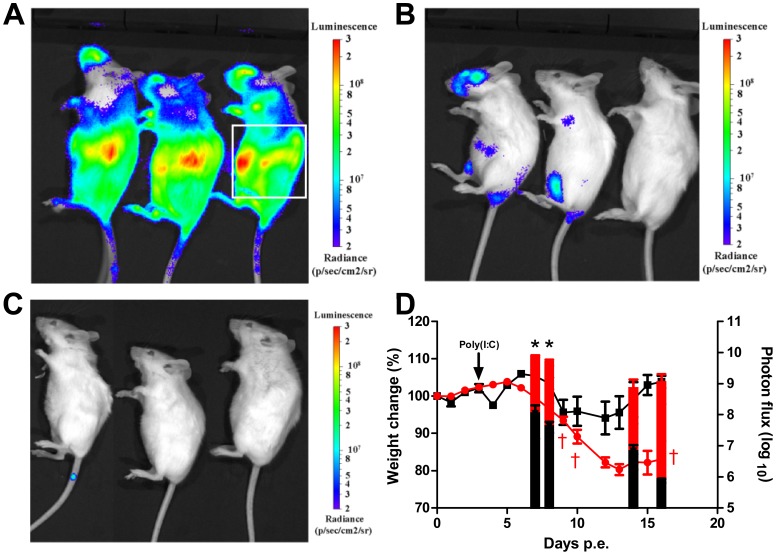
Influence of Poly(I:C) treatment on viral dissemination evaluated by *in-vivo* bioluminescence imaging. BALB/c mice were infected with 2 i.n. ECTV-Luc LD_50_ and left untreated (n = 5) or treated with poly(I:C) on day 3 p.e. (n = 3). (A) Infected untreated mice 8 days p.e. (B) Infected mice treated on day 3 p.e. with poly(I:C) and imaged on day 8 p.e. (C) Poly(I:C) treated group on day 3 p.e. imaged on day 16 p.e. Bioluminescent images were obtained using an f/stop of 1, binning factor of 4, and acquisition time of 1 sec (A, B) or 40 sec (C). Relative photon flux expression is represented by a pseudocolor heat map. (D) Morbidity, based on weight change (lines, left Y axis) and bioluminescence signal on days 7, 8, 14 and 16 p.e. (bars, right Y axis) of the groups shown in panels A–C (red for infected untreated, black for poly(I:C) treated on day 3 p.e.). Bioluminescent signal intensity as total photon flux (photon/s/cm2/sr), was calculated by region of interest (ROI) analysis on the chest and abdomen area marked by a white box on the right mouse in panel (A). Same ROI was used for all mice examined. Asterisk denote for significant reduction in photon flux (n = 3–5 in each group, P<0.05). Dagger represent dead mice.

To further elaborate on the mechanism of protection afforded by the adjuvants we assessed the effect of poly(I:C) on *in-vivo* dissemination of ECTV. BALB/c mice were infected intranasally with a lethal dose of ECTV expressing firefly Luciferase (ECTV-Luc, 2 LD_50_) and photon flux was determined using a bioluminescence imager (IVIS Lumina II). Data analysis revealed that within 7–8 days, ECTV-Luc efficiently disseminated to the lungs, liver and spleen, determined as the region of interest (ROI) (Figure 2 A). Bioluminescence analysis on days 7, 8 (Figure 2 B), 14 and 16 p.e. (Figure 2 C) revealed that poly(I:C) treatment on day 3 significantly reduced the signal intensity at the ROI by 2–3 logs compared to the signal in infected untreated mice (Figure 2 D, P<0.05 for days 7 and 8) in agreement with the improved protection induced by poly(I:C) in ECTV-Moscow infected mice (Table 1). Recovery following poly(I:C) treatment (day 16 p.e., Figure 2 C) was associated with gain of weight, improvement of animal condition and undetectable virus in the spleen (<75 pfu/spleen) and liver (<750pfu/liver). Even though bioluminescent signal from the lung was undetectable 16 days p.e., a very low viral load of 4.9×10^3^±3.0×10^3^ pfu/lung was still detected by plaque assays (Figure 2 D).

**Figure 3:**
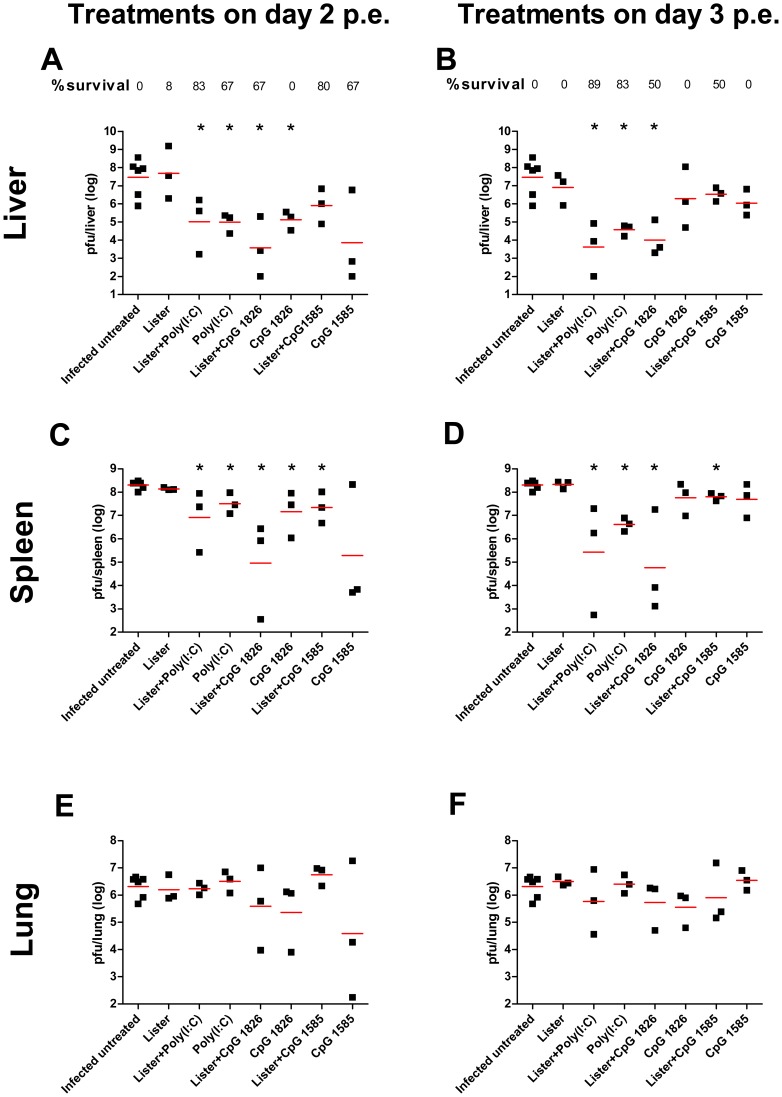
Viral load following postexposure treatments. Viral loads were determined by plaque assay from BALB/c mice 8 days postexposure (p.e.) following infection with 18 i.n. ECTV LD_50_. (A, C, E) viral load in livers, spleens and lungs of mice treated on day 2 p.e. (B, D, E) viral load in livers, spleens and lungs of mice treated on day 3 p.e. Horizontal lines represent the geometric mean of each group. Survival proportions of each group are designated. Asterisk denote for significant reduction in viral load (n = 3 in each treated group) compared to the infected untreated group (n = 6, P<0.05).

To further evaluate the efficacy of treatments, BALB/c mice were sacrificed on day 8 p.e. and viral loads in the lungs livers and spleens were determined. In the peripheral secondary organs, the viral load in the challenged untreated group reached 2.9×10^7^ pfu/liver and 2.0×10^8^ pfu/spleen (Geomean, Figure 3 A, C). VACV-Lister vaccination 2 or 3 days p.e. had no effect on the viral loads detected in the spleen or liver (Figure 3 A–D). Poly(I:C) treatments given with or without VACV-Lister vaccination on day 2 p.e. significantly reduced the viral load in the liver and the spleen (P<0.05 vs. infected untreated group). This reduction was further enhanced when Poly(I:C) treatment was given on day 3. Correlation between protection and reduced viral load in the spleen and liver was also found following co-administration of VACV-Lister vaccination and CpG 1826 whether given on day 2 or 3 (Figure 3 A–D). None of the treatments mentioned above reduced viral load in the lungs (Figure 3 E–F). Similar results were obtained in C57BL/6j mice (Figure S2).

### Effective Poly(I:C) treatment correlates with reduced serum IFNγ in advanced stages of the disease and prevention of tissue damage

**Figure 4:**
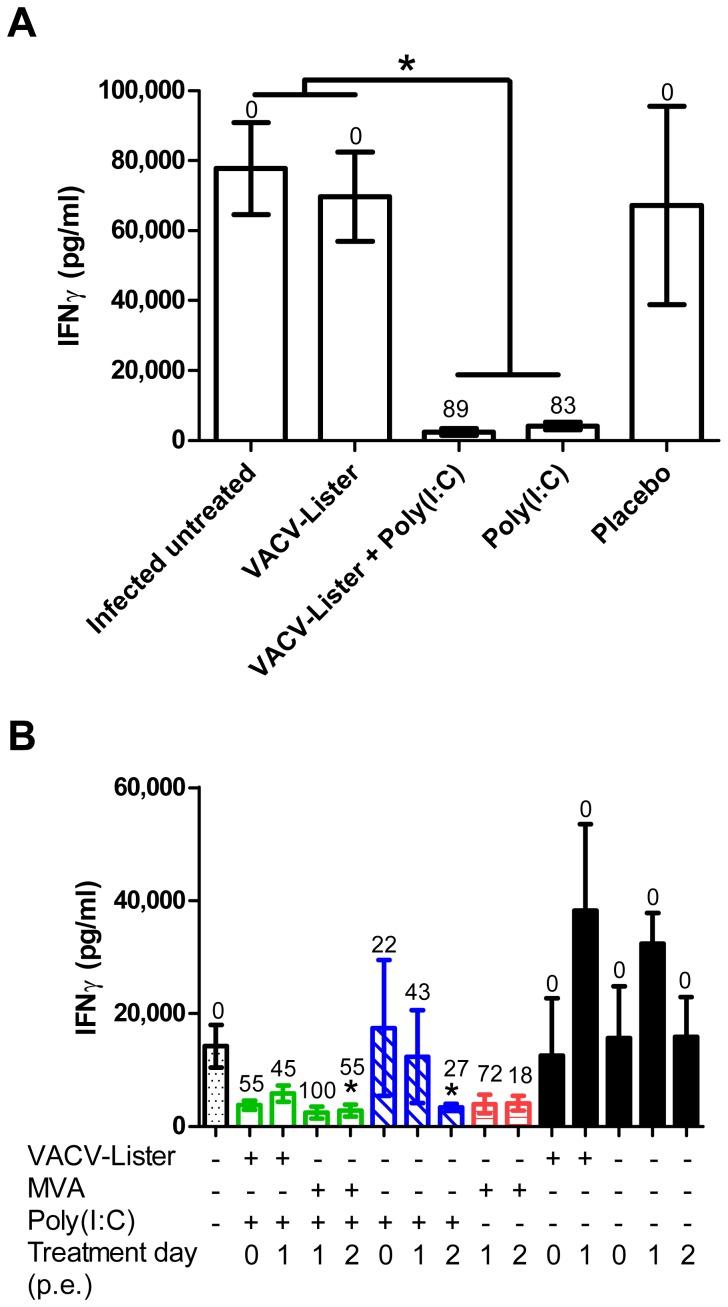
Changes in serum IFNγ levels following p.e. treatments. IFNγ levels were determined 8 days p.e. (A) BALB/c mice were infected with 5–18 i.n. ECTV LD_50_ and treated on day 3 p.e. with VACV-Lister (n = 6), VACV-Lister and Poly(I:C) (n = 6), Poly(I:C) (n = 6) or placebo (PBS, n = 3). Asterisk indicate for significant difference (P<0.05, n = 9 in the infected untreated group). (B) C57BL/6j mice were infected with 2–3 i.n. ECTV LD_50_ and treated on days 0–2 p.e. with Poly(I:C) with or without VACV-Lister or MVA and only vaccines treatments (n = 3–6 in each group). Asterisk indicate for significant difference in IFNγ levels compared to the infected untreated group (n = 9, P<0.05). For each experimental group percent protection values are designated above each bar (%) which refer to data collected from all similar treatment groups (Table 1 and 2). Naive BALB/c (n = 5) and C57BL/6j mice (n = 4) had IFNγ values of 11.9±1.0 pg/ml and 8.0±0.8 pg/ml, respectively.

Following lethal infection of non-human primates with variola virus, the level of IFN-gamma (IFNγ) increases at the advanced final stages of the disease to about 3 orders of magnitude over the cytokine level in naive animals [42]. We quantified the level of IFNγ in the sera of BALB/c mice collected on day 8 p.e. At this time point infected untreated mice were at the advanced stages of the disease based on their weight loss and viral load in different organs. While serum level of IFNγ in naïve mice was 11.9±1.0 pg/ml, in the infected untreated BALB/c mice the IFNγ levels reached 77,800±13,000 pg/ml (Figure 4 A). Similar levels were also detected in infected mice that were vaccinated with VACV-Lister or placebo treated. Administration of poly(I:C) on day 3 p.e., either alone or with the vaccine that conferred protection of 83% and 89% protection, respectively, resulted in significant reduction in the level of IFNγ(2,461±957 pg/ml for the VACV-Lister+poly(I:C), and 4,166±1,135 pg/ml for only poly(I:C), Figure 4 A, P<0.01 vs. infected untreated group).

Analysis of IFNγ in C57BL/6j mice revealed similar behavior to BALB/c, yet the magnitude of the response (changes in IFNγ) to ECTV infection and to protective treatments was more robust in the BALB/c mice (Figure 4 B). Combined treatments of VACV-Lister or MVA with poly(I:C) as well as administration of only poly(I:C) were associated with reduced IFNγ levels in accordance with the improved survival.

**Figure 5:**
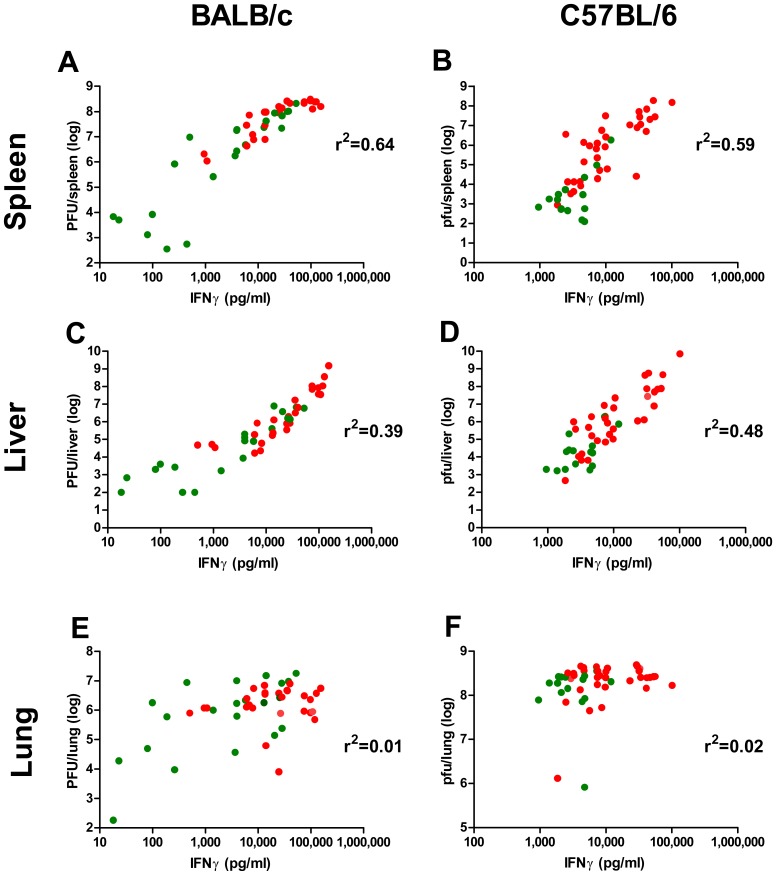
Correlation between IFNγ and viral load. (A, C, E) Viral loads and IFNγ levels in BALB/c mice infected with 18 i.n. ECTV LD_50_, untreated (n = 6) or single treated on day 2 or 3 with: poly(I:C) with or without VACV-Lister, CpG-ODNs 1585 and 1826 with or without VACV-Lister and VACV-Lister only (n = 3/group). (B, D, F) Viral loads and IFNγ levels in C57BL/6j mice infected with 2–3 i.n. ECTV LD_50_, untreated (n = 6) or single treated on day 0 with: poly(I:C) with or without VACV-Lister, VACV-Lister or placebo; day 1: poly(I:C) with or without VACV-Lister or MVA, VACV-Lister or MVA, placebo; day 2: poly(I:C) with or without MVA (n = 3/group), MVA or placebo (n = 3/group). (A, B) spleens; (C, D) livers and (E, F) lungs. Green dots - examined mice from groups in which the survival rate was 50% and above. Red dots - examined mice from groups in which the survival rate was less than 50%, (n = 48 for either BALB/c or C57BL/6j).

Serum IFNγ levels on day 8 p.e positively correlated with viral loads in the spleen and liver for each mice, in both mouse strains (Figure 5 A–D, treatments on days 2–3; R^2^ are indicated near each graph, P<0.0001 in all cases). Viral load in the lung, on the other hand, was not affected by the treatments and hence IFNγ levels in the serum did not correlate with lung viral load (Figure 5 E, F). In addition, the distribution of the range of IFNγ in BALB/c mice was wider than in C57BL/6j, allowing to draw thresholds that may allow to distinguish between a successful (<700 pg/ml) or an ineffective (>100,000 pg/ml) treatment.

**Figure 6:**
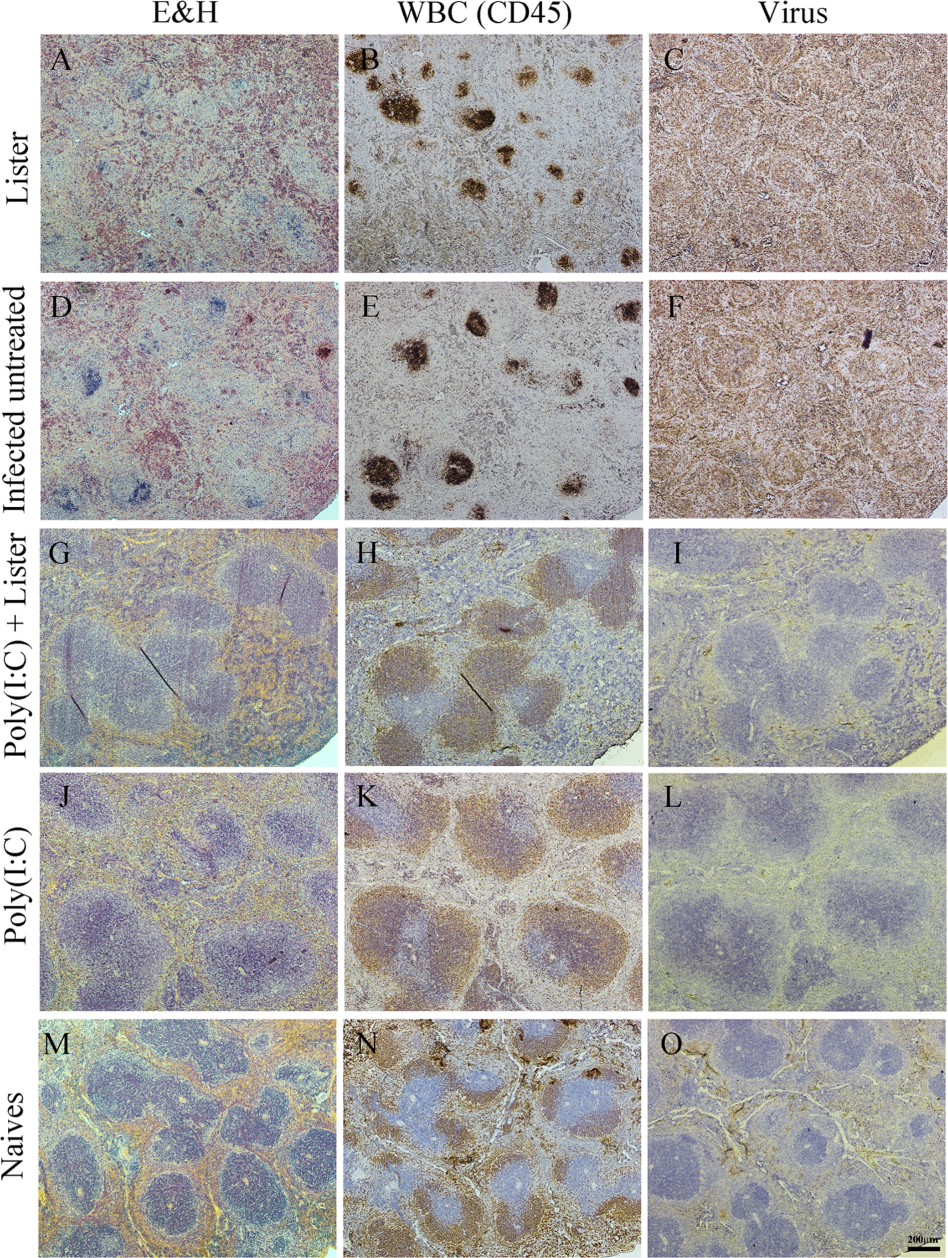
Effect of p.e. treatments on the spleen of infected mice. Spleens were dissected 8 days p.e. from treated mice (3 days post treatment) (A–C, G–L), from untreated (D–F) or from un-infected naïve mice (M–O). Left column - hematoxylin and eosin stain (H&E), middle column - CD45 (brown staining of WBC), right column – viral antigens (brown staining ). Serial sections of (A–C) VACV-Lister treatment; (D–F) infected untreated; (G–I) poly(I:C) and VACV-Lister treatment and (J–L) poly(I:C) treatment. Magnification in all images: X40.

To investigate the pathological effect of the infection on target organs we performed histopathological examination of spleens, livers and lungs of treated (on day 3 p.e.) vs. infected untreated mice on day 8 p.e. Spleens of infected untreated mice (or mice treated with only VACV-Lister) exhibited a severe tissue destruction and complete loss of tissue architecture. The white pulp was mostly destroyed and lymphocyte necrosis and presence of karyorrhexis were observed throughout the spleen (Figure 6 A, D). Lymphocytes (positively stained for CD45) appeared in the remnants of white pulps (Figure 6 B, E) while immunohistochemical sections were positively stained for ECTV viral antigens throughout the necrotic tissue (Figure 6 C, F). In contrast, spleens of mice treated with the combined poly(I:C) - VACV-Lister treatment showed normal morphology which was accompanied by a typical white pulp architecture, (positively stained with CD45) and undetectable viral antigens (Figure 6 G–I). Spleens of mice treated with poly(I:C) had the same morphology as in the combined treatment group (Figure 6 I–L) except for few small necrotic foci which were positive for viral antigens and negative for CD45 cells (Figure S3).

Histopathology examination was also carried out on livers of treated and untreated mice. While in the combined VACV-Lister – poly(I:C) and the poly(I:C) treated mice only a mild or moderate diffuse hepatocytic vacuolar degeneration was observed, in infected untreated and the VACV-Lister mice, livers exhibited multifocal hepatocytic necrotic foci throughout the liver and massive staining for ECTV (Figure S4). The lungs of the mice treated with poly(I:C) alone or combined with VACV-Lister had normal tissue morphology while mild signs of interstitial pneumonia were observed in the lungs of the infected untreated or only VACV-Lister treated group. In the Lister treated group an increased number of neutrophils were detected in the lungs (data not shown).

### Modulation of the adaptive immune response following Poly(I:C) administration

**Figure 7:**
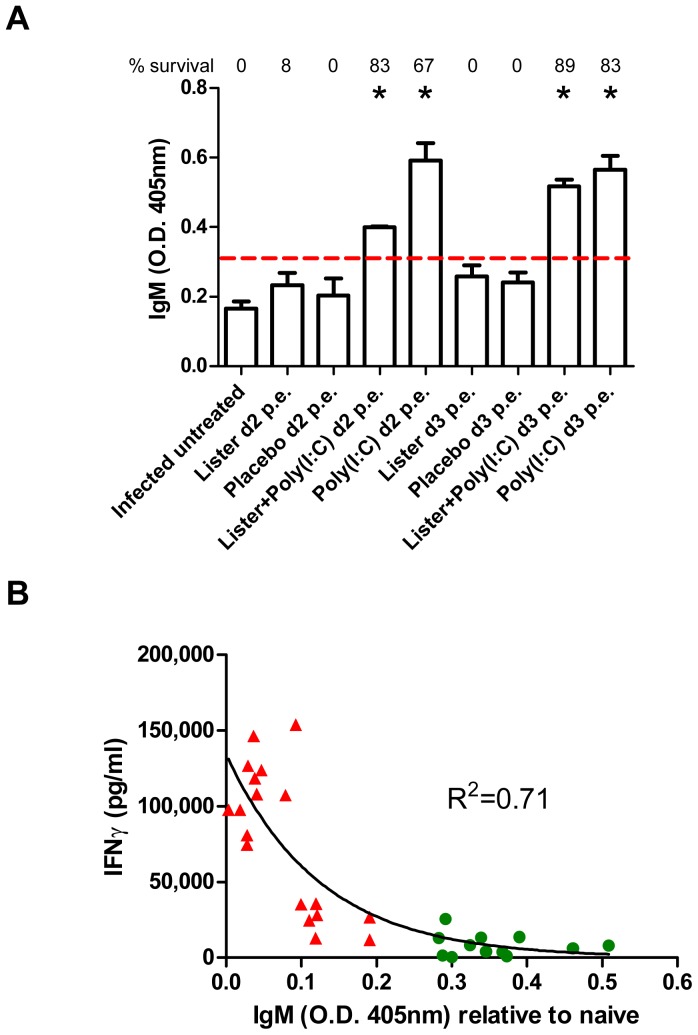
Correlation of IgM levels and treatment efficacy. (A) IgM level in the sera of BALB/c mice collected 8 days post i.n. infection with 18 LD_50_ of ECTV was determined by ELISA. Survival proportions of each group are designated above each bar. Asterisk denote for significant difference compared to the infected untreated group (P<0.01, n = 6 in the infected untreated group, n = 3 in the treated groups, *t*-test). Dotted line represent limit of detection. (B) Correlation between IFNγ and IgM levels of the mice presented in panel A. Green circles - examined mice from groups in which the survival rate was 50% and above. Red triangles - examined mice from groups in which the survival rate was less than 50%. R^2^ value represents a non-linear fit.

Having demonstrated that ECTV proliferation and spread to target organs is reduced following administration of poly(I:C), we further investigated the treatment effects on the immune response. Administration of poly(I:C) on day 2 or 3 p.e., induced higher levels of anti-poxvirus specific IgM in the sera of BALB/c mice when measured on 8 days p.e. (Figure 7 A, P<0.01 compared to the infected untreated group) regardless whether VACV-Lister was co-administered. IgM titers were below the limit of detection in infected untreated, placebo treated or VACV-Lister vaccinated mice. Solid protection (67% and above) were associated with high titer of IgM antibodies.

Since IFNγ production is known to abrogate immunoglobulin production (including IgM, [43], [44]), we examined whether the observed changes in IgM response correlated with the level of IFNγ (both examined from day 8 p.e. mice sera). Indeed, we found that high levels of IFNγ negatively correlated with the titers of poxvirus specific IgM (Figure 7 B, R^2^ = 0.71).

**Figure 8:**
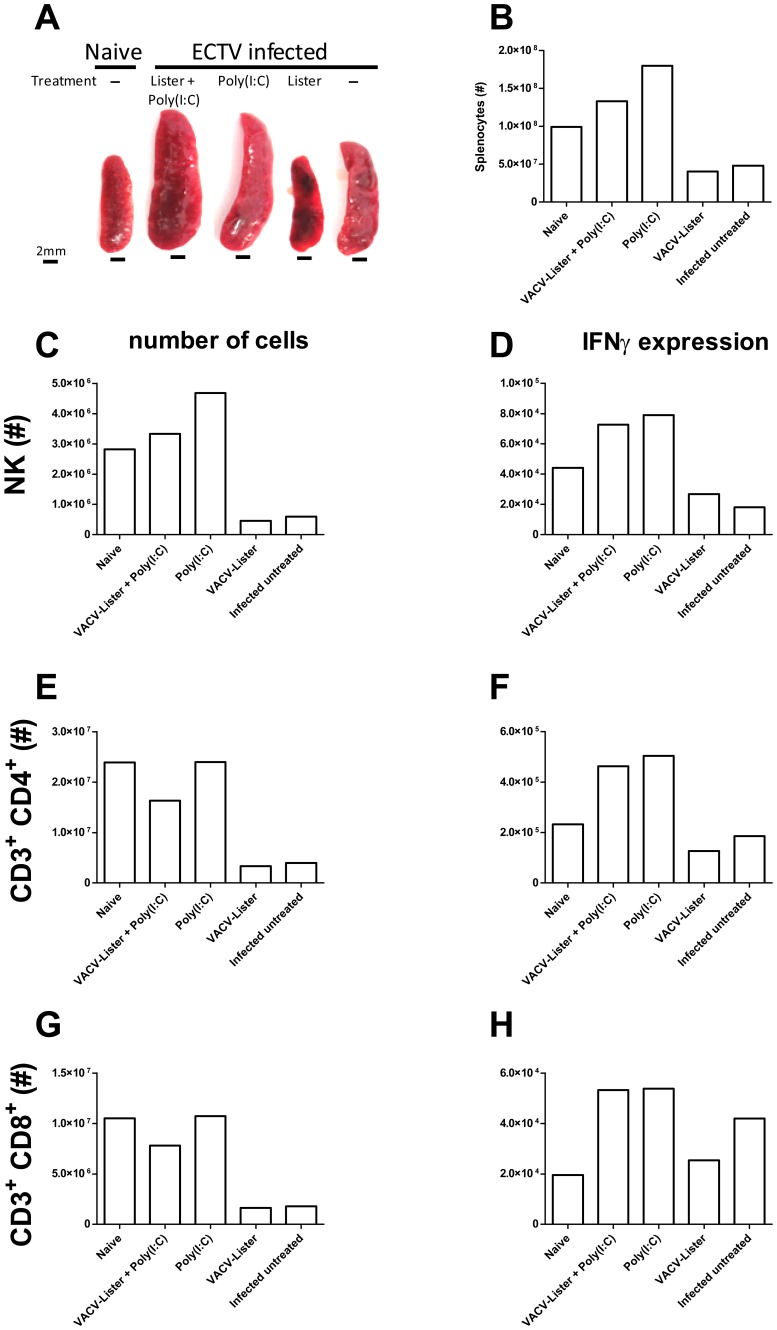
Cellular-immune response following p.e. treatment. BALB/c mice were infected with 4 i.n. ECTV LD_50_, left untreated or treated on day 3 p.e. and their spleens were photographed (A) and analyzed by flow-cytometry for the distribution and activation (intracellular IFNγ) of various cell populations. (B) Counting of viable lymphocytes was performed under light microscope. (C–H) Number of total and activated cells of the different cell populations: NK (C, D), of CD3^+^ CD4^+^ (E, F) and CD3^+^ CD8^+^ (G, H) cells, respectively.

To further characterize the cellular-immune response following p.e. adjuvant administration, BALB/c mice were inoculated with a lethal dose of ECTV (4LD_50_), administered poly(I:C) on day 3 and the effect was quantified based on the number and activation of natural killer (NK) as well as CD4^+^ and CD8^+^ T cells on day 8 p.e. (i.e. 5 days after treatment). Gross morphology comparison revealed enlarged spleens in infected mice treated with poly(I:C), with or without VACV-Lister, while spleens of infected mice vaccinated with VACV-Lister or left untreated, appeared smaller and had a mosaic non-homogeneous appearance in contrast to spleens of mice that were administered with poly(I:C) that appeared normal (Figure 8 A). Indeed, the number of total lymphocytes in the relatively smaller spleens was lower than in spleens of naive mice, whereas poly(I:C) treatment correlated with increased number of lymphocytes per spleen (Figure 8 B). The reduction in total number of lymphocytes was accompanied by reduction in the numbers of NK cells, CD3^+^CD4^+^ and CD3^+^CD8^+^ T cells. (Figure 8C, E, G , respectively). In contrast, poly(I:C) treatment resulted in an increase in the number of NK cells (Figure 8 C). Further analysis of lymphocyte activation revealed that poly(I:C) treatment induced activation of NK cells, CD3+CD8+ and CD3+CD4+ T-cells as depicted by intracellular staining for IFNγ (Figure 8 D, F, H). Whereas lymphocyte activation in poly(I:C) treated mice involved both CD4 and CD8 T-cells as well as NK cells, untreated mice or VACV-Lister vaccinated mice exhibited differential activation of CD3+CD8+ T-cells only. Thus, solid protection was associated with an orchestrated activation of CD3^+^CD8^+^, NK and CD3^+^CD4^+^ cells.

### Poly(I:C) contributes to protection via TLR3 and induction of type 1 IFNα

**Figure 9:**
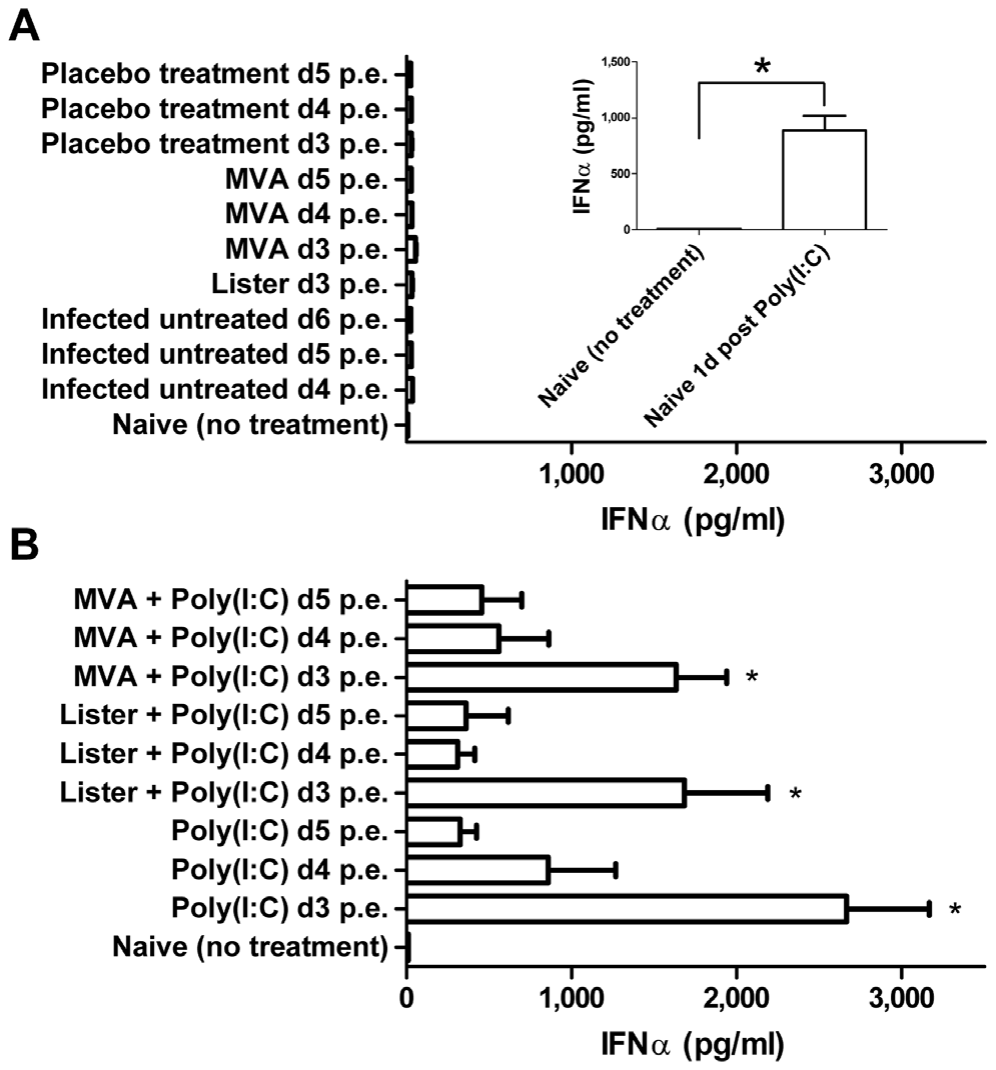
Elevation of IFN-α levels following Poly(I:C) administration. IFN-α levels were examined in BALB/c mice sera. (A inset) Sera of uninfected naïve mice before (n = 2) and 24 h after poly(I:C) administration (n = 6). IFN-α in sera of 20 i.n. LD_50_ ECTV (A) infected untreated mice examined on days 4 (n = 6), 5 (n = 3) or 6 (n = 3) p.e. or 24 hours after treatment with VACV-Lister given on day 3, MVA on day 3, 4 or 5 or placebo treated on days 3, 4 or 5 (n = 3 in all groups). (B) IFN-α in sera of mice 24 hours after treatment with poly(I:C) on days 3, 4 or 5 given alone or in combination with VACV-Lister or MVA (n = 3–5/group).

Binding of poly(I:C) to TLR3 leads to receptor activation and synthesis of type I IFNs. In order to evaluate the antiviral immune response following poly(I:C) administration we quantified the effect of poly(I:C) administration on IFNα production by measuring its concentration in the sera of uninfected naïve mice. Poly(I:C) administration resulted after 24 hours in a significant three order of magnitude increase in the level of serum IFNα, (888.1 pg/ml vs. 8.5 pg/ml, P<0.01, Figure 9 A, Inset).

Since ECTV infection affects host immunity including type I IFN response [37], we determined the serum levels of IFNα in infected mice following treatments given 3, 4 or 5 days p.e. (Figure 9 A, B). IFNα level remained low (<54 pg/ml) in ECTV infected mice that were untreated or vaccinated with VACV-Lister or MVA (Figure 9 A). In contrast, poly(I:C) administration, with or without the vaccines, resulted in elevated IFNα levels ranging between 310 and 2,660 pg/ml (Figure 9 B). Poly(I:C) treatments, with or without the vaccine, given on day 3 p.e. induced significantly higher IFNα levels compared to the infected untreated group at the same time point (day 4 p.e., P<0.01, Figure 9 A, B). Co-administration of VACV-Lister or MVA maintained the ability of poly(I:C) to induce IFNα. The induction of IFNα was associated with improved statistically survival in these groups (Table 1). Disease progression interfered with the ability of poly(I:C) to induce IFNα, resulting in reduced IFNα response on day 4 or 5 compared to day 3 (Figure 9 B).

Finally, we investigated whether induction of IFNα by poly(I:C) was sufficient to protect mice against subsequent ECTV infection. To this end, mice were administered with poly(I:C) one day before or on the day of exposure (20 ECTV i.n. LD_50_). No protection was achieved during these early treatments. Poly(I:C) treatments in this experiment, given on days 1, 2 or 3 conferred protections of 50%, 83% and 100%, respectively (data not shown), suggesting that efficient protection requires the orchestrated immune modulation effect of poly(I:C) in conjunction with sufficient amount of viral antigens.

## Discussion

The success of smallpox eradication allowed for the gradual discontinuation of the world-wide vaccination campaign, which consequently resulted in a gradual increase in the percentage of the unprotected population. Thus, in the case of accidental or deliberate VARV release, there will be a need for a rapid and effective treatment which could be delivered to as many people as possible in a timely manner. Smallpox in humans is associated with a prolonged incubation period (7–17 days in natural infections) thus allowing time for p.e. vaccination or treatment. Based on our experience in mice, the longer the incubation period, the better the effect of the treatment and the recovery rates [8].

In this report we presented the effect of TLR agonists on the therapeutic window of p.e. smallpox vaccination in mice. Following infection, the innate immune system provides the host with a first line of defense followed by an adaptive immune response. This rapid innate response is attributed amongst others to detection of foreign components by TLRs, leading to activation of various pathways, including antiviral immunity, T cell stimulation and elevated inflammation signals [15], [17]. Poxviruses have developed numerous strategies to subvert/interfere with the host immune response (Reviewed by [37]). We have recently showed that active vaccination up to 3 days p.e. confer efficient protection necessitating the use of a high dose of the vaccine (1×10^8^ pfu in mice) [8]. This study also demonstrated that although not required, the type I IFN pathway (IFNAR signaling) plays a role in p.e protection by vaccination. In addition, Samuelson and her colleagues demonstrated that TLR9 is not required, yet, plays a role in p.e. protection by active vaccination against ECTV [36].

These facts led us to examine the effect on the immune response by activating two TLRs; TLR3 and TLR9 using their synthetic agonists (synthetic double stranded RNA molecules and bacterial CpG DNA, respectively). While adjuvants are routinely added to inactivated or subunit vaccines to confer pre-exposure immunity [32], [45]–[47], in the present study we co-administered the adjuvant with a live vaccine and examined it as a therapeutic/p.e. treatment. Indeed, a single treatment combined of VACV-Lister and poly(I:C) conferred solid protection even on day 5 p.e. while treatment with VACV-Lister alone (at the conventional low vaccine dose) was efficient only up to day 1 p.e. (Table 1, [8]). In the current study, the TLR3 agonist Poly(I:C) conferred better protection than the TLR9 agonists. This may reflect a major role for TLR3 in protection against ECTV and/or a higher potency of poly(I:C) compared to the ODN's in activating the immune response. Poxviruses encode a variety of immune modulators known to interfere with the antiviral immune response [37]. Whether the antiviral immune response following DNA administration (ODN's) is inhibited by immune evasion mechanisms encoded by ECTV more efficiently than antiviral activities directed against dsRNA has not been covered by us.

Following poly(I:C) administration, profound activation of type 1 IFN response (IFNα) was detected, followed by activation of humoral and cellular immunity. Significantly IFNα was not induced by ECTV infection suggesting that immune evasion by ECTV might be interfering with the induction of IFNα. Even p.e. vaccination with VACV or MVA did not result in activation of IFNα, further supporting the idea that ECTV infection effectively blocks the induction of type 1 IFN response. The ability of ECTV to reduce the level of IFNα is demonstrated in Figure 9 B showing that delaying poly(I:C) administration from day 3 to 5 result in reduced IFNα response. Indeed EVM166 of ECTV, the ortholog of the B18R soluble type 1 IFN binding protein, effectively binds and excludes the mouse type 1 IFN from the circulation which contributes to the virulence of ECTV [48]. In addition to ECTV, the vaccine strains (Lister and MVA) used in this study also encode the orthologs of B18R however in a p.e. vaccination scenario, the effect of the B18 encoded by VACV-Lister or MVA is probably masked by EVM166. We have previously demonstrated by vaccination of IFNAR knockout mice [8], that in the absence of IFN α/β receptor, vaccination with MVA is protective, but the mice lose more weight than the parental strain (C57BL/6j), indicating that at least MVA can induce protective type 1 IFN response even if EVM166 is functional. In the present study, p.e. vaccination with VACV-Lister or MVA in conjunction with poly(I:C) could not alleviate the immune suppression induced by ECTV at late stages of the disease. (Figure 9 B). This clearly demonstrates the advantage of the co-administered poly(I:C) that even at late stages of the disease (e.g. day 5) induces sufficient amount of IFNα that escapes binding of the viral IFN binding proteins and accounts for protective immunity. In addition to the soluble IFN binding proteins, poxviruses encode multiple proteins that interfere with IFN pathway at multiple levels, which can effectively interfere with type 1 IFN response [37].

Interestingly, moribund mice with high viral burden in the lung, spleen and liver that eventually succumb to infection had significantly higher level of serum IFNγ than mice that received protective treatments (poly(I:C) +/− vaccine; Figures 3–5). Secretion of IFNγ, mainly from activated NK and T cells, has immune regulatory functions including activation of macrophages and neutrophils, enhancement of NK cell killing activity, regulation of B and T cell responses to antigens, stimulation of specific cytotoxic T cells, promotion of chemokine and cytokines expression as well as contribution to the protection against viral pathogenesis [49]–[51]. Indeed, viral exposure which confers protective immunity (such as dermal poxvirus exposure in mice) results in transient induction of IFNγ response [7], [52]. However, upon intranasal lethal exposure, robust and sustained IFNγ response is detected at late stages of the disease correlating with pathological damage to tissues and death. This high concentration of IFNγ has an inhibitory effect on the adaptive immune response [43], [44] as demonstrated by the significant inhibition of IgM production, (Figure 7). Indeed, the phenomenon of high concentration of IFNγ associated with pathological damage to tissues at late stages of the disease, referred to as a “cytokine storm”, has been reported by Jahrling et al. in VARV infected non-human primates [42]. The linear correlation between elevated level of serum IFNγ and viral load has been demonstrated also in RSV infected mice [53] further indicating that induced IFNγ response can be associated either with protective immunity or with immune pathology depending on the timing and context of the disease. Holechek *et. al.* [54] reported recently that p.e. vaccination with VACV expressing IFNγ protects mice against VACV and ECTV infection and attributed the protection to the early expression of mouse IFNγ as discussed above. Since overt late expression of IFNγ can be pathologic while early expression has protective value, the therapeutic use of IFNγ should be addressed using transient rather than constitutive IFNγ deficiency models. Based on the data presented in this work, it will be interesting to evaluate a recombinant vaccinia virus expressing IFNα as an intrinsic adjuvant for p.e. treatment as was recently carried out with IFNγ. The treatment efficacy of repetitive doses of IFNα and IFNγ against vaccinia and monkeypox viruses in mice further indicates to the therapeutic potential of modulating the immune response in treatment of orthopoxvirus infections [49], [55].

Following protective treatments (poly(I:C)+/−vaccine) viral loads in the spleen and liver were significantly reduced on day 8 compared to unprotected mice. However, no statistical significant difference between the groups was detected when comparing the lungs, regardless of treatment efficacy, suggesting that viral clearance from the lungs at later stages of the disease is not a direct result of the antiviral activity of IFNα, rather protection is achieved by the immunomodulatory effects of IFNα and other immune mediators/cells, somewhat similarly to what was reported by Holechek et. al. [54]. Since there is no data suggesting sustained ECTV replication at the lungs upon recovery, we non-invasively monitored the level of ECTV from day 8 to 16 p.e. using virulent ECTV-Luc. Indeed, recovery was associated with viral clearance from the liver and spleen while low level of lung ECTV was still detected even 16 days p.e. in mice that otherwise appeared healthy. Thus, viral clearance from the lungs is not a prerequisite for recovery and the underlying mechanism allowing recovery while viable viruses still exist at the lung awaits further clarification. Whether gradual virus control at the lung favors recovery since robust anti-viral response is known to have pathological consequences, or whether the immune response cannot achieve rapid viral clearance in the lung unlike the spleen and liver, is still unknown.

The cellular immune response in the spleen, 8 days p.e., differed between mice receiving poly(I:C) or left untreated. Poly(I:C) administration was associated with a higher number of NK cells and coordinated activation of NK and T cells (CD8^+^ and CD4^+^), which in conjunction with activation of IgM antibody production correlated with improved protection. Infected-untreated mice, showed profound activation of CD8^+^ killer cells while both CD4^+^ and NK cells were poorly active, indicating of an imbalanced immune response, unlike mice that were administered with poly(I:C) with or without vaccines. While activated CD8^+^ cells are required for protection by pre-exposure vaccination with VACV against lethal poxvirus infection [40], [56], [57], the coordinated activation of additional cells (e.g. NK and CD4^+^ T cells) should be important in regulation of the response. The robust elevation of IFNγ levels in the serum of unprotected animals as a clear indication of immune pathology rather than immune protection, as previously shown for VARV infection of nonhuman primates [42].

It has been previously demonstrated that in B-cell deficient mice, MVA vaccination 2 days pre-exposure conferred rapid protection [40]. Similarly, pre-exposure vaccination of B-cell deficient mice with VACV-Lister conferred protection against lethal ECTV intranasal exposure (our unpublished data) suggesting that antibody production is not essential for rapid protection or pre-exposure against ECTV infection. However, p.e. immunization with either VACV-Lister or MVA was not protective in B-cell deficient mice (our unpublished data) pointing to the important role B-cells in a p.e. scenario. Based on the correlation between IgM production and survival and the pleiotropic effects of poly(I:C) that results in activation of a variety of immune cells, it remains to be elucidated whether poly(I:C) would mount protective immune response in the absence of either B or T cells.

Pre-exposure immunization with VACV-Lister or MVA and even rapid immunization with MVA [40], [58] induces robust virus specific CD8+ immune response, required for protection against ECTV infection. Interestingly, 6 days post lethal intranasal ECTV exposure of C57BL/6 mice, unvaccinated mice show robust virus specific CD8+ (B8R_20–27_) response but later on deteriorate and succumb to infection (our unpublished data). At that time point, their CTL response resembles the response following protective vaccination, suggesting that while CTL response is necessary for protection, the complicated and imbalanced immune response following exposure fails to protect even though virus specific CTL response was initially induced. Whether the mechanism of protection afforded by poly(I:C) includes also elevated number of virus specific CD8+ cells early p.e., activation of a modified repertoire of epitope specific CD8+ cells or modulation of the immune response including activation of virus specific CD4+ cells and modulation of other T cell types (e.g. Th17 and T-regs) remains to be elucidated.

Our data indicates the importance of timing in a single treatment regime. Whereas p.e. administration of poly(I:C) (days 3–5) effectively induced IFNα secretion and protected against ECTV infection, pre-exposure or even short-term (up to day 1 p.e.) administration, induced IFNα secretion but was unable to confer protection, highlighting the requirement for viral antigens to achieve protection. We have previously demonstrated that in p.e. vaccination, the antigenic mass of the vaccine determines the protection efficacy [8]. We showed that p.e. administration of the adjuvant poly(I:C) (up to day +4 in BALB/c mice) allows to achieve protective immunity without the need to supply high-dose vaccination. Unless co-administered with a vaccine, the efficacy of poly(I:C) administration depends on the antigenic mass of the infecting virus (ECTV) as discussed above. However, at later stages of the disease (Day +5), we assume that co-administered vaccine was required to achieve sufficient protection since at this stage of disease, immune evasion mechanisms interfere with the ability of poly (I:C) to exert its adjuvanting role. This was demonstrated by the decreased ability of poly(I:C) to induce IFNα at late stages of disease (Figure 9 B).

When translated to humans, due to the long incubation period in smallpox, in a case of p.e. ring vaccination, it will be unclear who was exposed and when. As a result, a “therapeutic window of adjuvant only” cannot be determined, necessitating the co-administration of vaccine to achieve protection in a mixed population composed of exposed, suspected and naive individuals. A similar phenomenon in which some treatments given on the first day were less effective compared to treatments administered latter on was demonstrated for p.e. therapy with Cidofovir (CDV) and CMX001 [7], [10]. The reduced efficacy of CDV at early days is attributed to its rapid clearance from the circulation, resulting in the unavailability of the drug at late stages of the disease. Similarly to poly(I:C), single CDV treatment was most efficient when given not before day 2 up to day 5 or 7 (depending on the dose). Similarly to CDV, preliminary experiments of repetitive treatments with poly(I:C) aiming to increase the bioavailability of poly(I:C) showed promising results, (unpublished data).

The efficacy of p.e. administration of adjuvants in human smallpox is unknown, yet, the longer incubation period compared to mousepox, is expected to result in comparable protection rates. This approach has potential implications also for conventional anti smallpox pre-exposure vaccination. The two types of vaccines used in our work (VACV-Lister and MVA) are known for their highly efficacy. VACV-Lister was one of the vaccines used around the world to eradicate smallpox and MVA is a modern highly attenuated vaccine which should have fewer post-vaccinal side effects due to its poor ability to replicate in mammalian hosts. One of the drawbacks of MVA is the dose needed (prime and boost, each 1×10^8^ pfu) which is significantly higher than the single VACV-Lister dose (1×10^6^ pfu). Addition of an adjuvant, such as poly(I:C) or CpG-ODN, might improve the vaccination efficacy and might allow to achieve comparable protection with a reduced vaccine dose. Applying the same strategy for VACV-Lister might reduce the possibility or the severity of post-vaccinal complications and in the case of MVA, can change the protocol to a single vaccination (without the need for a booster) or reduce the vaccination dose (i.e. 1×10^6^ instead of 1×10^8^ pfu). If vaccination with reduced doses would prove efficacious, it would simplify the mass production of MVA. A comparison between the conventional regimes and the suggested ones with the addition of adjuvants are currently under examination.

In conclusion, adjuvants based on synthetic RNA and DNA given in conjunction with smallpox vaccines, VACV-Lister or MVA, improved significantly the efficacy of p.e. protection compared to the vaccines alone. Beneficial effect of the RNA adjuvant, poly(I:C), was observed even without co-vaccination but only up to a certain time point. Improved protection was associated with accelerated humoral and cellular immune responses and resulted in prevention of mortality, alleviation of the disease symptoms and reduced viral load in target organs. This novel approach of combining adjuvants with a live vaccine in a p.e. regime can potentially be utilized for other viral diseases in which p.e. treatment is relevant.

## Supporting Information

Figure S1 **Morbidity based on weight change following post exposure (p.e.) treatments of C57BL/6j mice.** Mice were infected with 2–3 i.n. ECTV LD_50_. (A) Poly(I:C) treatments with or without VACV-Lister and only VACV-Lister treatment on day 0 p.e. (B). Poly(I:C) treatments with or without VACV-Lister or MVA and only vaccines treatments on day 1 p.e. (C) Poly(I:C) treatments with or without MVA and only MVA treatment on day 2 p.e. Asterisk denote for significant difference in the area-under-the curve of weight changes along the entire experiment of the treated groups vs. the infected untreated group (* P<0.05, ** P<0.01, *** P<0.001, **** P<0.0001, *t*-test). Data collection for each treatment (weight change (mean, SE)) is indicated. Mortality out of the total mice number in each group is designated color coded next to the legend.
(TIF)

Figure S2 **Viral load following p.e. treatments.** Viral load in livers (A), spleens (B) and (C) lungs (C) of C57BL/6j mice was determined by plaque assay. The organs were analyzed 8 days post infection with 2 i.n. ECTV LD_50_. Mice were treated on days 0–2 p.e. as indicated. Horizontal lines represent the geometric mean of each group. Survival proportions of each group are designated. Asterisk denote for significant reduction in viral load (n = 3 in each treated group) compared to the infected untreated group (n = 6, P<0.05).
(TIF)

Figure S3 **Alleviation of damage to the spleen by poly(I:C) treatment.** Spleens were taken from mice 8 days p.e. treated on day 3. (A, B) Hematoxylin and eosin stain (H&E). (C, D) Anti vaccinia stain for ECTV detection (positive stain in brown). (E, F) CD45 for WBC stain (positive stain in brown). Figures B, D and F are enlargements of the boxes presented in A, C and E respectively. Magnification for images A, C, E: X40; B, D, F: X1000.
(TIF)

Figure S4 **Effect of poly(I:C) treatment on the liver of ECTV infected mice.** Livers were taken from mice 8 days p.e. treated with poly(I:C) on day 3 (A–F), infected untreated (G–H) or not infected naïve mice (I–J). Left column – Hematoxylin and eosin and stain (H&E), right column – anti vaccinia stain for ECTV detection (positive stain in brown). Serial sections of (A, B) poly(I:C) and VACV-Lister treatment; (C, D) poly(I:C) treatment and (E, F) VACV-Lister treatment. Magnification in all images: X40.
(TIF)
