# Supplementary figures and images for: TLR3 and TLR9 Agonists Improve Postexposure Vaccination Efficacy of Live Smallpox Vaccines
Source: PLoS One. 2014 Oct 28;9(10):e110545. doi: 10.1371/journal.pone.0110545 (PMC4211728; doi:10.1371/journal.pone.0110545)

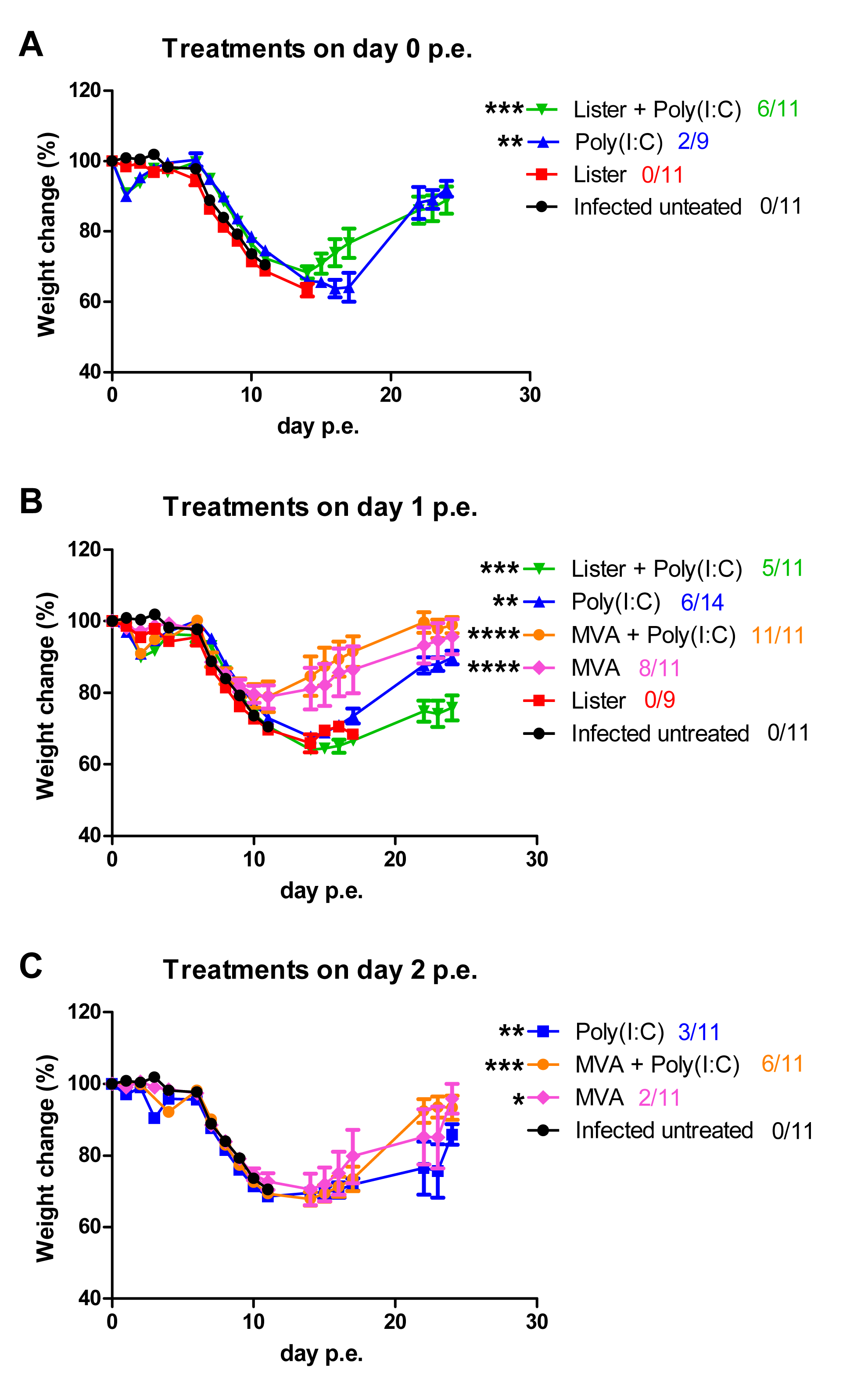

Supplement: Figure S1 — Morbidity based on weight change following post exposure (p.e.) treatments of C57BL/6j mice. Mice were infected with 2–3 i.n. ECTV LD50. (A) Poly(I:C) treatments with or without VACV-Lister and only VACV-Lister treatment on day 0 p.e. (B). Poly(I:C) treatments with or without VACV-Lister or MVA and only vaccines treatments on day 1 p.e. (C) Poly(I:C) treatments with or without MVA and only MVA treatment on day 2 p.e. Asterisk denote for significant difference in the area-under-the curve of weight changes along the entire experiment of the treated groups vs. the infected untreated group (* P<0.05, ** P<0.01, *** P<0.001, **** P<0.0001, t-test). Data collection for each treatment (weight change (mean, SE)) is indicated. Mortality out of the total mice number in each group is designated color coded next to the legend. (TIF) [file pone.0110545.s001.tif]

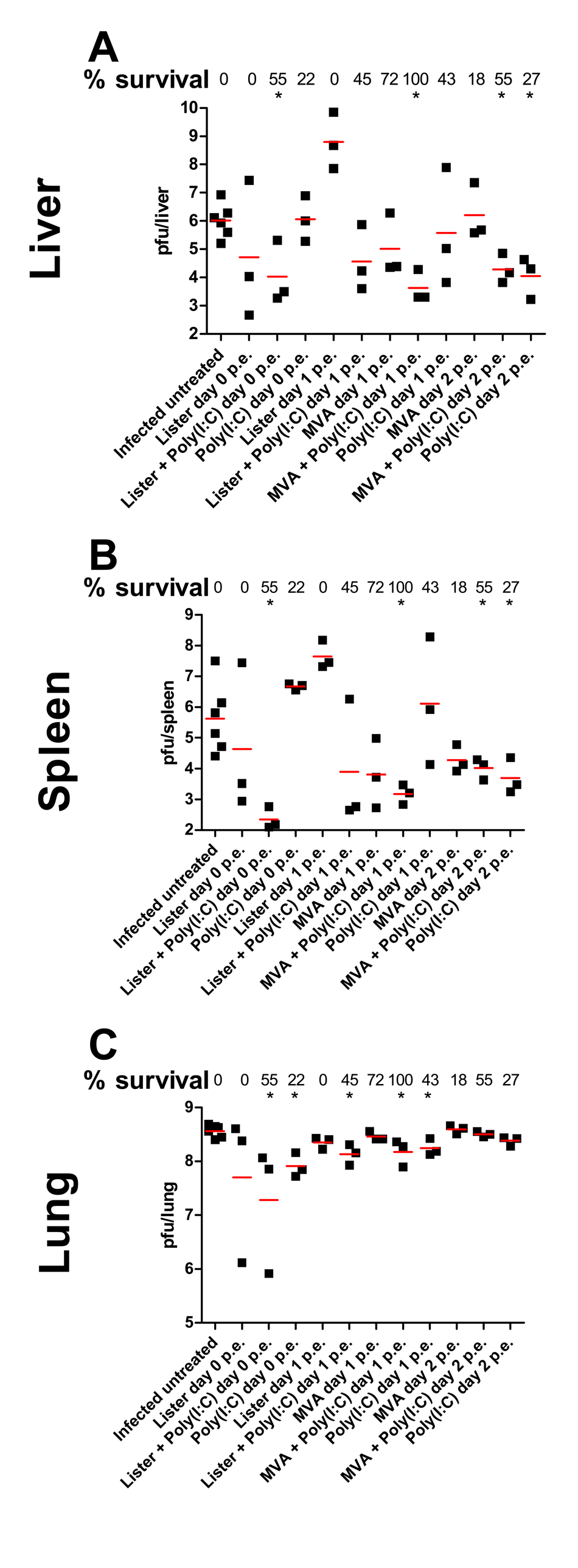

Supplement: Figure S2 — Viral load following p.e. treatments. Viral load in livers (A), spleens (B) and (C) lungs (C) of C57BL/6j mice was determined by plaque assay. The organs were analyzed 8 days post infection with 2 i.n. ECTV LD50. Mice were treated on days 0–2 p.e. as indicated. Horizontal lines represent the geometric mean of each group. Survival proportions of each group are designated. Asterisk denote for significant reduction in viral load (n = 3 in each treated group) compared to the infected untreated group (n = 6, P<0.05). (TIF) [file pone.0110545.s002.tif]

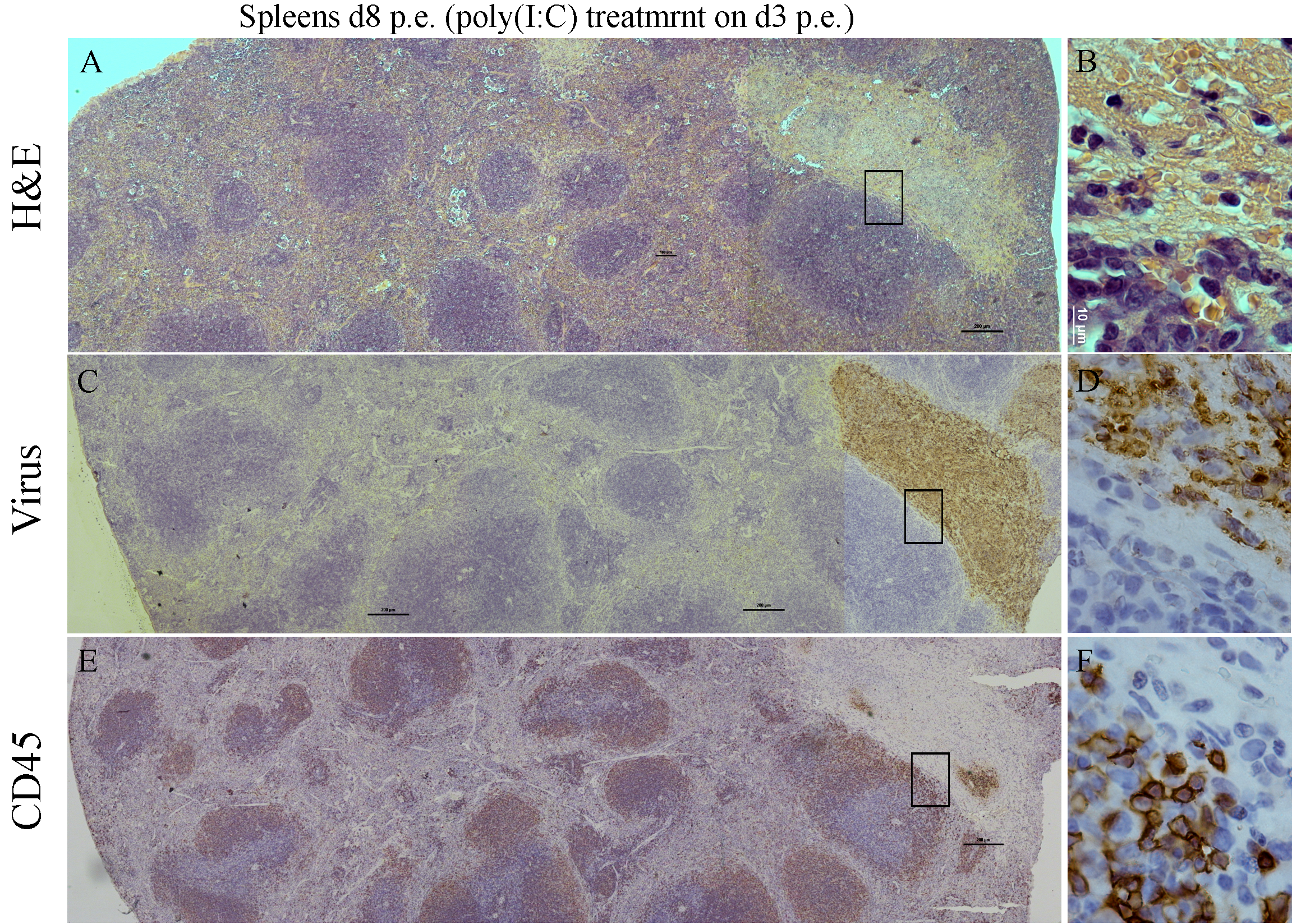

Supplement: Figure S3 — Alleviation of damage to the spleen by poly(I:C) treatment. Spleens were taken from mice 8 days p.e. treated on day 3. (A, B) Hematoxylin and eosin stain (H&E). (C, D) Anti vaccinia stain for ECTV detection (positive stain in brown). (E, F) CD45 for WBC stain (positive stain in brown). Figures B, D and F are enlargements of the boxes presented in A, C and E respectively. Magnification for images A, C, E: X40; B, D, F: X1000. (TIF) [file pone.0110545.s003.tif]

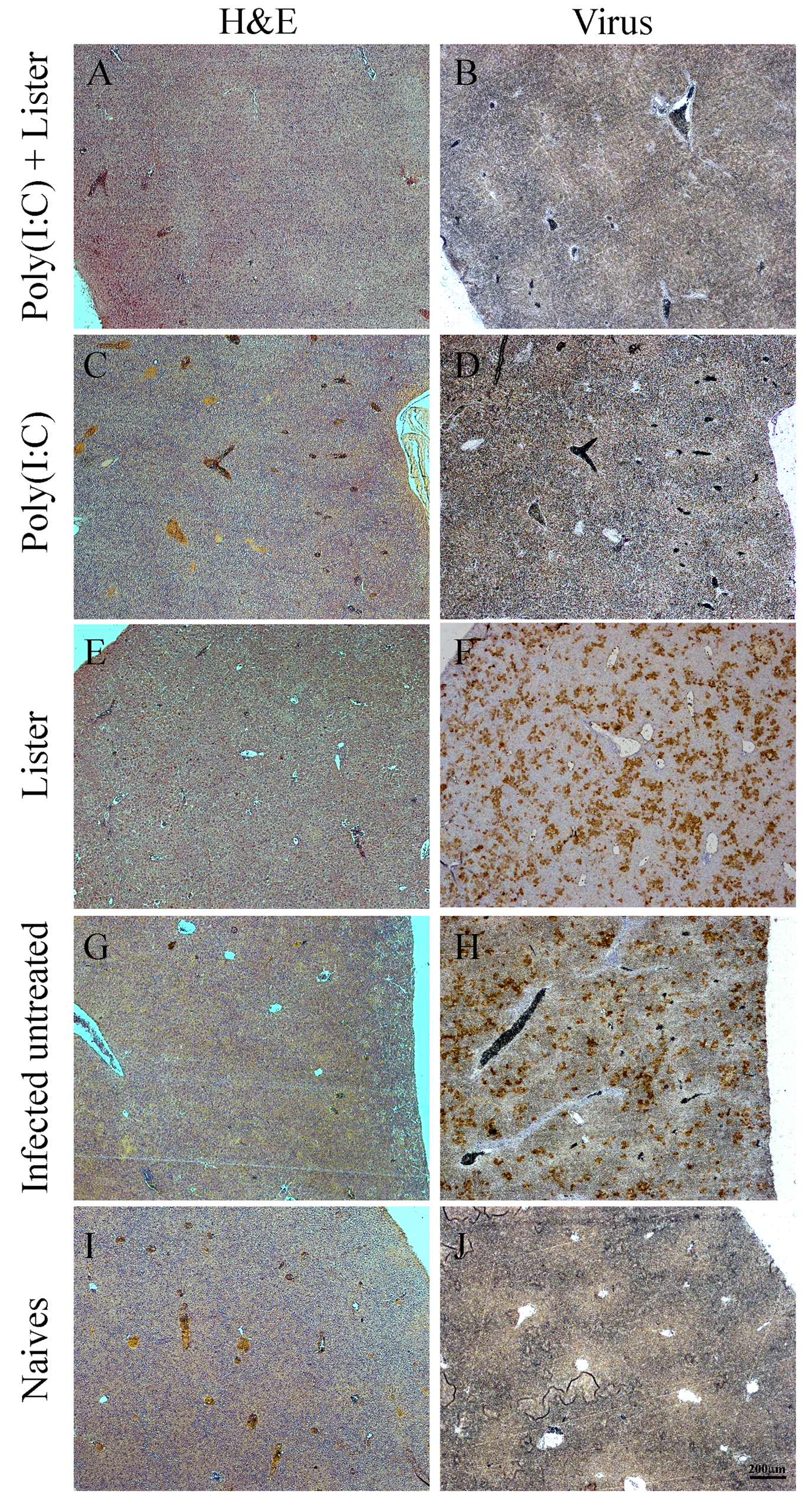

Supplement: Figure S4 — Effect of poly(I:C) treatment on the liver of ECTV infected mice. Livers were taken from mice 8 days p.e. treated with poly(I:C) on day 3 (A–F), infected untreated (G–H) or not infected naïve mice (I–J). Left column – Hematoxylin and eosin and stain (H&E), right column – anti vaccinia stain for ECTV detection (positive stain in brown). Serial sections of (A, B) poly(I:C) and VACV-Lister treatment; (C, D) poly(I:C) treatment and (E, F) VACV-Lister treatment. Magnification in all images: X40. (TIF) [file pone.0110545.s004.tif]
